# Elevated virus infection of honey bee queens reduces methyl oleate production and destabilizes colony-level social structure

**DOI:** 10.1073/pnas.2518975122

**Published:** 2025-10-14

**Authors:** Alison McAfee, Abigail Chapman, Armando Alcazar Magaña, Katie E. Marshall, Shelley E. Hoover, David R. Tarpy, Leonard J. Foster

**Affiliations:** ^a^Michael Smith Laboratories, Department of Biochemistry and Molecular Biology, University of British Columbia, Vancouver, BC V6T1Z4, Canada; ^b^Department of Applied Ecology, North Carolina State University, Raleigh, NC 27607; ^c^Life Sciences Institute, Department of Biochemistry and Molecular Biology, University of British Columbia, Vancouver, BC V6T1Z3, Canada; ^d^Department of Zoology, University of British Columbia, Vancouver, BC V6T1Z4, Canada; ^e^Department of Biological Sciences, University of Lethbridge, Lethbridge, AB T1K3M4, Canada

**Keywords:** queen–virus interactions, pheromones, lipid trafficking, supersedure, reproduction

## Abstract

Pathogenic threats to reproductive individuals pose a profound challenge to the stability of insect societies. In honey bees (*Apis mellifera* L.), severe virus infections in queens can trigger worker-initiated supersedure, a socially coordinated replacement of the queen that, while risky, is essential when her reproductive competence is compromised. How viruses impact the physiology of queen hosts, who bear unique reproductive burdens within their colonies, and how this perturbs colony social order remains poorly understood. We hypothesized that the supersedure response is mediated by pathogen-induced, intensity-dependent changes in queen pheromonal signaling. Laboratory infection experiments revealed that queens challenged with deformed wing virus B and black queen cell virus infections demonstrated a reduction in methyl oleate, a key component of the queen retinue pheromone, and field data corroborated this association. Lipidomics analysis demonstrated that infection coincides with a systemic lipid deficiency, especially in triacylglycerides (major energy reserves), providing a physiological link among viral stress, ovarian atrophy, and altered pheromone output. Notably, artificial suppression of ovary investment via restricted laying also caused methyl oleate production to decline; therefore, high virus infection likely indirectly suppresses methyl oleate production by reducing ovary mass. In field trials, we further show that synthetic pheromone blends containing methyl oleate significantly suppressed queen cell rearing compared to no-pheromone controls, whereas blends lacking this compound yielded an intermediate effect. These results demonstrate that virus-induced reproductive decline disrupts pheromone signaling, revealing a plausible mechanistic pathway by which pathogens can erode social cohesion.

## Significance

As the mother to tens of thousands of individuals within their colonies, honey bee queens bear the enormous burden of egg laying, but the reproductive cost of pathogenic infections is poorly understood. Here, we show that elevated virus infections—ubiquitous in most beekeeping operations—shrink the queen’s ovaries, which causes a change in her pheromone production. That pheromone shift signals to the workers that the queen is compromised and needs to be replaced, triggering queen cell rearing (supersedure). These data provide a working mechanism for initiation of queen replacement and illustrate how virus–host interactions within an individual can cause an adaptive change in pheromone-coordinated behavior within the colony, ultimately resulting in rearing a new, healthy queen.

As the sole reproductive female in a colony of tens of thousands, the honey bee (*Apis mellifera*) queen has an enormous reproductive output, the demise of which is frequently among the most commonly reported causes of colony losses (1–5). In healthy queens, this reproductive feat is enabled by the nutritious diet supplied to her by workers (facultatively sterile females) in addition to physiological and behavioral specialization [reviewed by Fèvre et al. (6)]. During the seasonal period of peak colony growth (normally late spring), the queen lays approximately 850 to 3200 eggs per day, the average total weight of which is greater than her own body (7–9). Such reproductive division of labor, one of the hallmarks of eusociality (10), means that the queen does little in the colony other than walk, lay eggs, eat royal jelly provided by the workers, and secrete pheromones that maintain social cohesion (11–13). The nutritious royal jelly diet—originating from the workers’ hypopharyngeal and mandibular glands (14) —is rich in proteins, lipids, and carbohydrates (15, 16), providing the queen with all the resources required to enable nearly continuous egg laying and pheromone production.

Each mature oocyte is approximately 1.4 to 1.6 mm long (17) and is provisioned with sufficient nutrients during oogenesis, which is carefully regulated by molecular “checkpoints” (18), to enable the embryo to develop over the subsequent 72 h postlaying. Similar to other insects (19), approximately 40% of the dry mass of oocytes is composed of lipids (20), nearly all of which are originally supplied to the queen in the form of fatty acids within royal jelly (in addition to what may be biosynthesized from carbohydrates in the queen’s fat body) (16, 21). Metabolic pathways and, in particular, lipid trafficking in queens are therefore expected to be highly specialized processes that can support rapid turnover from nutrient inputs to egg outputs.

Nutrients can enter oocytes developing in any of the queen’s ~360 ovarioles (22) by at least three distinct mechanisms: nurse cell dumping of cytoplasmic contents, receptor-mediated endocytosis, and enzyme-mediated delivery (19, 23), with lipids primarily entering via the latter two routes as lipoprotein cargo (19). Vitellogenin, apolipophorin-III (ApoLP-III), and apolipophorins-I/II (ApoLP-I/II) are the major lipoproteins in insects (24, 25), and while they can all function as lipid carriers, vitellogenin and ApoLP-III are also pathogen-associated molecular pattern (PAMP) binding proteins with roles in insect immunity (26–28). Vitellogenin’s main immunological role is to facilitate transgenerational immune priming by binding and transporting PAMPs into oocytes (29), whereas ApoLP-III appears to act as a humoral immunity potentiator [reviewed by Weers and Ryan (24)]. Vitellogenin is an inefficient lipid carrier (on a per protein basis), containing only 8 to 10% lipids in its loaded form (19), and is responsible for transferring only about 5% of the oocyte’s total lipid content (30) from the hemolymph to the oocyte via receptor-mediated endocytosis (31). Lipid-loaded ApoLP-I/II is a high-density lipoprotein (HDL; 30 to 50% lipid) and can also be taken up by oocytes via receptor-mediated endocytosis (19); however, when ApoLP-I/II associates with loaded ApoLP-III particles, it forms a low-density lipoprotein (LDL) complex. These LDLs are responsible for delivering most (~90%) of oocytes’ lipids, where entry occurs via enzyme-mediated delivery (in which the lipids, but not the protein, are taken up by the oocyte) (30).

Though they are long-lived reproduction specialists, honey bee queens are still susceptible to many of the same pathogens that are abundant in adult workers, including viruses (32, 33). Given the enormous challenge of managing the *Varroa destructor* parasite and the viruses it vectors in contemporary beekeeping (34), combined with frequent reports of poor queen quality (2–5), we argue that queen virus infections are likely an underappreciated problem for honey bee colony management. In our previous research, we found that experimental infections of queens with either Israeli acute paralysis virus (IAPV) or a combination of deformed-wing virus-B (DWV-B) and black queen cell virus (BQCV) can cause a reduction in the queens’ ovary mass (i.e., a small-ovary phenotype) and this compromises their ability to lay eggs (33, 35, 36). Since immune stimulation alone was sufficient to reduce ovary mass, this implies that a resource allocation trade-off between reproduction and immunity underlies, at least in part, the small-ovary phenotype (35); however, the mechanism of such a trade-off is still unknown, as is whether the trade-off occurs in a bidirectional manner (i.e., is a reduction in ovary mass sufficient to increase investment in immunity?). Although many studies in insects have shown that increased fecundity is linked to poorer immunity [reviewed by Schwenke et al. (37)], the nature of this relationship is often complicated by life stage transitions that confound with fecundity (38–45), and reproductive investment has not been experimentally reduced to test for reversibility.

Interestingly, ApoLP-III could mediate a reproduction–immunity trade-off, since research in other insect species suggests its immune-stimulating and lipid transport functions are mutually exclusive (46), and it appears to only function as an immune stimulator in its lipid-bound conformation (47–49). We speculate that, upon associating with PAMPs, ApoLP-III may be unable to participate in HDL complexation and thus stymie the main route of lipid entry to oocytes. Likewise, when ovary investment is restricted and fewer lipids are being shuttled to oocytes, this may make more noncomplexed ApoLP-III particles available to bind PAMPs. These ideas remain to be tested.

Over time, a honey bee queen’s fecundity eventually wanes, at which time the workers will initiate supersedure cell rearing to replace the existing queen (50). This activity poses a necessary risk to the colony, with colonies exhibiting such “queen events” being 3.1 times more likely to perish in the subsequent 50 d (51). The mechanism by which workers recognize queen failure and commit to the task of supersedure is not known, but since previous research has identified correlations between queen fertility characteristics and pheromone abundance (52–56), and pheromone extracts from the queen’s mandibular glands partially suppress queen rearing (57, 58), changes in queen pheromones could be involved. The honey bee queen retinue pheromone (QRP) is a synergistic blend of at least nine distinct compounds—9-oxo-2(E)-decenoic acid (9-ODA), 9-R- and 9-S-hydroxydec-2(E)-enoic acid (9-HDA), 4-hydroxy-3-methoxyphenylethanol (homovanillyl alcohol; HVA), methyl p-hydroxybenzoate (methylparaben; HOB), methyl (Z)-octadec-9-enoate (methyl oleate; MO), (Z9,Z12,Z15)-octadeca-9,12,15-trienoic acid (linolenic acid; LEA), hexadecan-1-ol (palmityl alcohol; PA), and (E)-3-(4-hydroxy-3-methoxyphenyl)-prop-2-en-1-ol (coniferyl alcohol; CA)—some of which originate from the mandibular glands (9-ODA, 9-HDA, HVA, HOB, and CA) while others are present elsewhere in the body (MO, LEA, and PA) (12).

The complete QRP blend elicits numerous and profound physiological and behavioral effects on drones (males) and workers (12, 59). Two competing theoretical frameworks explaining how such effects are realized are the “queen control hypothesis” (whereby the queen manipulates the workers via her pheromones) and the “honest signal hypothesis” (whereby pheromones convey information about the queen’s quality, allowing workers to evaluate the benefit of continued investment in her care) (60). Both scenarios are compatible with a change in pheromonal signaling mediating the initiation of supersedure to some degree—a decline in one or more pheromones or their components could either release the workers of queen control, or communicate to workers that the queen’s quality is no longer sufficient to support the colony’s population.

We previously observed that colonies headed by queens with more severe virus infections (i.e., higher cumulative loads of DWV-B, DWV-A, BQCV, and SBV) were more likely to rear supersedure cells (35). Combined with our findings that multiple viruses [IAPV (33) and a combination of DWV-B and BQCV (35)] can cause reductions in queen ovary mass, this suggests that elevated virus infections are generally harmful to queen quality. Here, we build on these findings by analyzing newly acquired and previously published lipidomics (55) and proteomics (61) data in combination with mechanistic trials to understand the systems underlying virus infection, ovary investment, pheromonal signaling, and the initiation of supersedure. We used an established method (55) to conduct lipidomics analysis on head extracts from experimentally infected queens to test the hypothesis that virus infection is associated with a decline in one or more QRP components. Indeed, high virus infection did reduce the QRP component methyl oleate (MO), and field data in which queenless colonies were exposed to pheromone blends including or excluding MO show that the presence of this compound contributes to inhibiting supersedure cell rearing. Given the lipid demands of egg laying (19) and that elevated virus infections interfere with ovary investment (33, 35), we also tested and provide support for the hypothesis that virus infection is associated with broad lipidomic changes. A change in pheromone abundance could be a direct result of infection or an indirect ovary effect; therefore, to disentangle these relationships, we evaluated QRP profiles in queens whose ovary masses were manipulated via laying restriction (55), and found that MO was indeed suppressed. Analysis of publicly available immune effector and major lipoprotein (vitellogenin, ApoLP-III, and ApoLP-I/II) data from these queens (61) enabled us to test whether the reproduction–immunity trade-off we previously observed functions in reverse—that is, whether restricting ovary mass is sufficient to boost immune effector abundance—and if abundance of circulating ApoLP-III is consistent with a potential role as a mediator. These data collectively provide a candidate mechanism by which workers recognize and respond to changes in queen quality induced by elevated virus infections and highlight unexpected ways that viruses can alter the physiology of eusocial reproductive hosts.

## Results

### Elevated Virus Infection Suppresses Methyl Oleate Abundance.

**Fig. 1.**
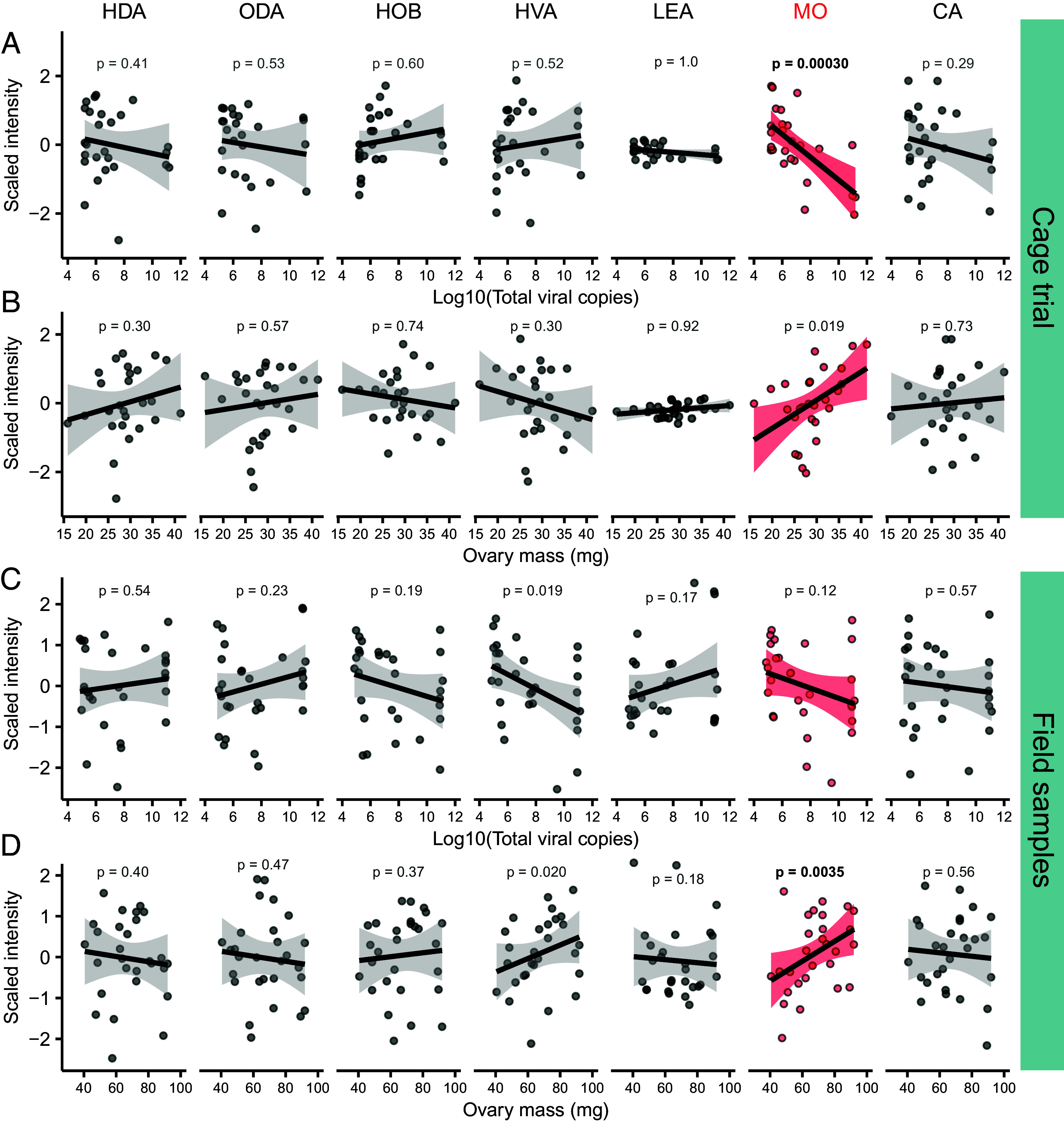
The QRP component methyl oleate is linked to queen virus infection and ovary mass. HDA = 9(R)-hydroxydec-2(E)-enoic acid; ODA = E-9-oxodec-2-enoic acid; HOB = methyl p-hydroxybenzoate; HVA = 4-hydroxy-3-methoxyphenylethanol; LEA = linolenic acid; MO = methyl oleate; CA = E-3-(4-hydroxy-3-methoxyphenyl)-prop-2-en-1-ol. Methyl oleate (red) was the only compound exhibiting significant correlations with tested variables (α = 0.0071; Bonferroni correction on 7 tests, bold text indicates *P* < α). Data were modeled using simple linear regressions. (*A*) Compound intensities in relation to total viral load (cage trial; N = 27 queens). (*B*) Compound intensities in relation to ovary mass (cage trial). (*C*) Compound intensities in relation to total viral load (field samples; N = 29 queens). (*D*) Compound intensities in relation to ovary mass (field samples).

We measured seven components of the queen retinue pheromone (QRP) blend in queen head extracts and found that, among queens with experimentally manipulated BQCV and DWV-B levels in our previously published cage trial (35), methyl oleate was the only QRP compound significantly linked to virus infection (F_1,24_ = 17.7, estimate = −0.33, *P* = 0.00031, α = 0.0071, Bonferroni correction; Fig. 1*A*). Methyl oleate was also positively associated with ovary mass (though the relationship was marginally not significant; F_1,25_ = 6.3, estimate = 0.08, *P* = 0.019, α = 0.0071, Bonferroni correction; Fig. 1*B*), as expected given that we previously established that queen viral load and ovary mass are inversely related (33, 35). Among queens heading colonies in the field, no QRP components were significantly linked to viral load, but one compound, again methyl oleate, was significantly positively linked to ovary mass (F_1,27_ = 10.2, estimate = 0.032, *P* = 0.0035, α = 0.0071, Bonferroni correction; Fig. 1 *C* and *D*).

### Methyl Oleate Inhibits Queen Cell Rearing.

**Fig. 2.**
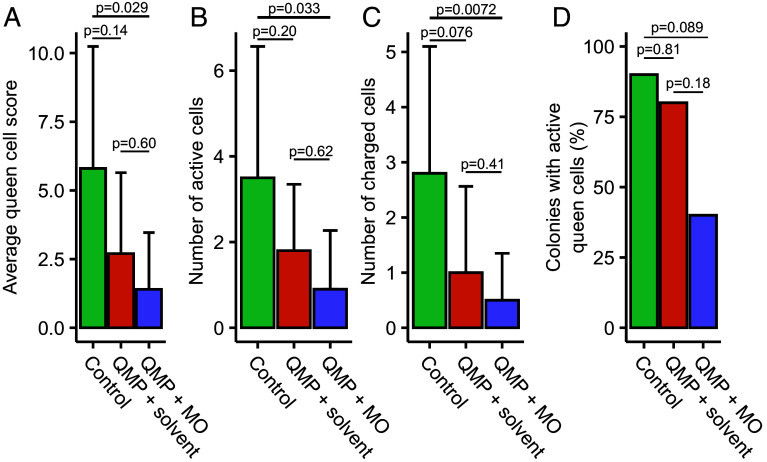
Methyl oleate suppresses queen cell rearing in combination with queen mandibular pheromones. Queenless colonies (N = 10 each) received one of three experimental treatments: control (no pheromone), QMP, or QMP + methyl oleate (MO). QMP = queen mandibular pheromone (a commercial blend including 9-ODA, 9(R)-HDA, 9(S)-HDA, HVA, and HOB). Average queen cell score represents an additive metric in which fully committed queen cells (charged cells with royal jelly and a larva) were weighted twice as much as cells with an egg or cells primed with royal jelly (but with no egg or larva). All three types of queen cells are referred to as “active cells”. Post hoc Tukey-adjusted *P* values are shown. (*A*–*C*) Statistical analysis was conducted using a generalized linear mixed model (specifying a Poisson distribution) with exposure group as a fixed effect and original colony source as a random effect. Error bars represent the SD. (*D*) Presence/absence of queen cells was modeled using the same model structure but specifying a binomial distribution.

To test whether methyl oleate is necessary to inhibit workers from rearing new queens, we conducted a field trial in which queenless colonies were exposed to either no pheromone, queen mandibular pheromone (QMP, which is composed of 9-ODA, 9(R)-HDA, 9(S)-HDA, HVA, and HOB, but not methyl oleate) or QMP + methyl oleate. As expected, colonies in the no pheromone group had the highest number of active queen cells (cells that were primed with royal jelly, contained an egg, or contained a larva and royal jelly), highest number of fully charged queen cells (i.e., contained a larva and royal jelly), the highest queen cell score (an additive metric that weights fully charged queen cells more heavily than primed or egg-containing queen cells), and the highest likelihood of having active queen cells (Fig. 2 *A*–*D*). All metrics were significantly reduced in colonies receiving QMP + methyl oleate (number of active cells: estimate = 1.4, z = 3.0, Tukey-adjusted *P* = 0.0066; charged cells: estimate = 1.8, z = 3.0, Tukey-adjusted *P* = 0.0072; cell score: estimate = 1.5, z = 3.2, Tukey-adjusted *P* = 0.0040) except for the likelihood of having active queen cells, which had a marginally nonsignificant reduction (estimate = 2.6, z = 2.1, *P* = 0.089). Colonies receiving QMP alone, however, tended to yield lower numbers of charged cells than the control group, but not significantly so (estimate = 1.1, z = 2.2, Tukey-adjusted *P* = 0.076), and the number of active cells, cell score, and likelihood of having active cells remained unchanged (number of active cells: estimate = 0.69, z = 1.8, Tukey-adjusted *P* = 0.18; cell score: estimate = 0.85, z = 2.0, Tukey-adjusted *P* = 0.12; likelihood of having active cells: estimate = 0.81, z = 0.62, *P* = 0.81). Since QMP + methyl oleate significantly reduced cell rearing metrics but QMP alone did not, methyl oleate appears to possess activity as a cell-rearing inhibitor in combination with other pheromone components.

### Elevated Virus Infection Causes Triacylglycerol Deficiencies.

**Fig. 3.**
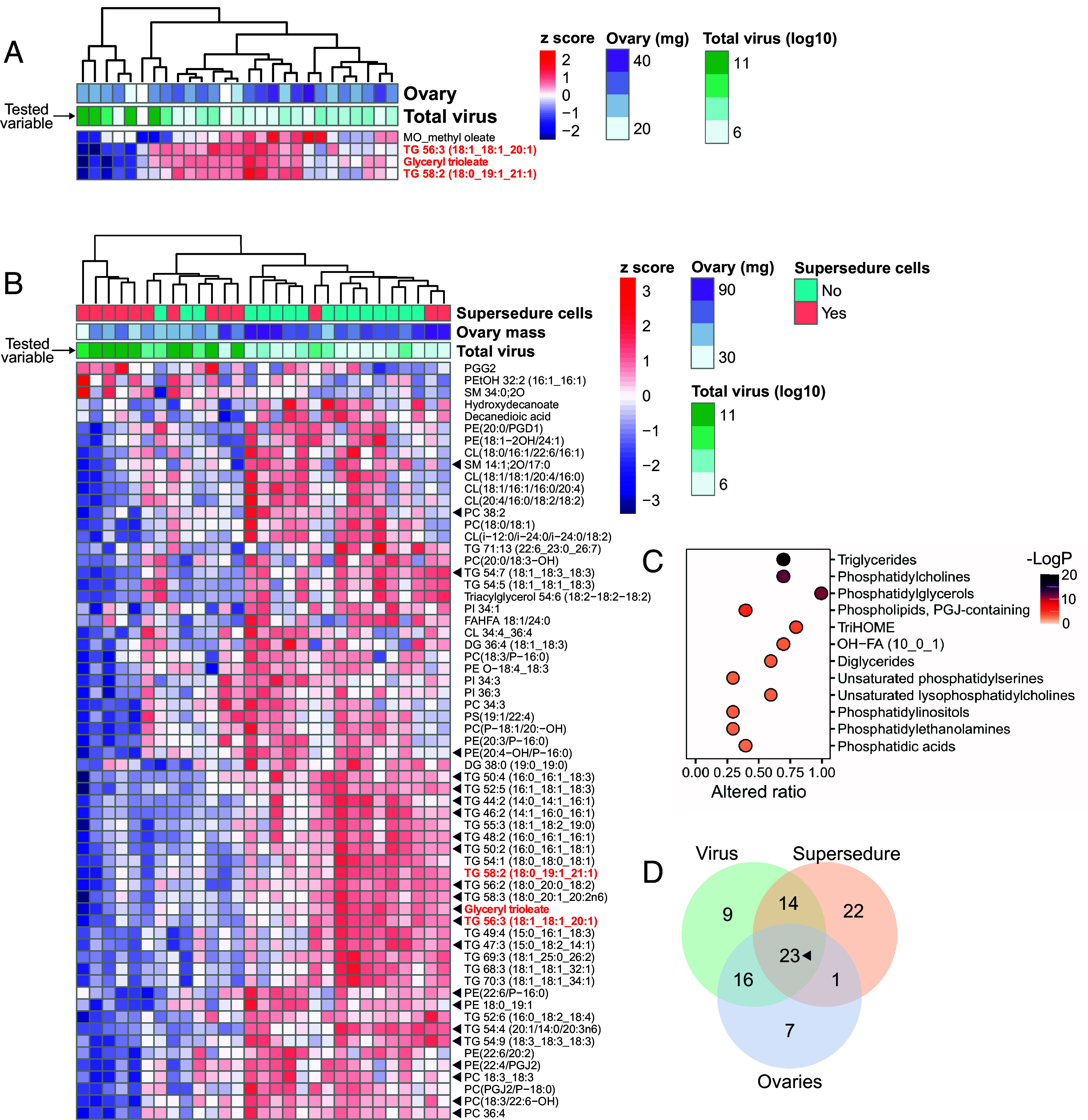
Annotated lipids significantly linked to virus infection. False discovery rates were controlled to 5% FDR (Benjamini–Hochberg correction). Differentially abundant compounds that are significant in both datasets (cage trial and field samples; N = 27 and 29 queens, respectively) are shown in red. (*A*) 4 out of 336 annotated compounds were significantly linked to virus infection in the cage trial data. (*B*) 62 compounds were significantly linked to virus infection in the field samples, 23 of which (black triangles) were also significantly linked to ovary mass and the presence of supersedure cells. (*C*) Significantly enriched structural classes (5% FDR, Benjamini–Hochberg correction) in the field sample data. (*D*) Overlap of annotated lipids significantly linked to total viral load, presence of supersedure cells, and ovary mass.

To assess broader lipidomic changes related to queen virus infection, we conducted differential abundance testing of all annotated lipids evaluated in queen head extracts from the cage trial and field samples. These data were acquired in concert with the pheromone analysis described above, providing insight into systemic effects of virus infection in addition to further pheromonal and nonpheromonal compounds that could respond to, or signal, infection status. Across both datasets, we identified 1,993 high-quality molecular features, of which 336 were annotated lipids, and 41 (12%) were triacylglycerols (Dataset S1). Principal component (PC) analysis shows that samples weakly cluster according to total viral load in the cage trial, as indicated both visually (*SI Appendix*, Fig. S1*A*) and by regressing total viral load against PC1 (estimate = −0.070, t = −1.9, *P* = 0.071) and PC2 (estimate = −0.098, t = −1.7, *P* = 0.094). No interaction between PC1 and PC2 was found, and the whole model (total virus ~ PC1+PC2) was marginally nonsignificant (F_2,22_ = 3.3, *P* = 0.055). The field samples, however, clustered more strongly according to total viral load (*SI Appendix*, Fig. S1*B*), with both PC1 (estimate = −0.16, t = −4.2, *P* = 0.00029) and PC2 (estimate = 0.12, t = 2.8, *P* = 0.010) being significant predictors using the same model structure (whole model: F_2,26_ = 12.6, *P* = 0.00015; again, no interaction was detected). Reflecting the PC analysis, only 4 out of 336 annotated lipids were significantly linked to total viral load among the cage trial queens, including methyl oleate as previously mentioned (FDR = 5%, Benjamini–Hochberg correction; Fig. 3*A*). The additional lipids were all triacylglycerols (glyceryl trioleate, TG 56:3, and TG 58:2), and all were negatively linked to viral load. In the queens sampled from the field, a larger number (62) of annotated lipids were significantly linked to viral load (FDR = 5%, Benjamini–Hochberg correction; Fig. 3*B*). Among these, only three increased with viral load, including a prostaglandin (PGG2), phosphatidylethanol (PEtOH 16:1 _ 16:1), and sphingomyelin (SM 34:0;2O). All other compounds were downregulated with virus abundance, including 25 (61%) of the annotated triacylglycerols. Structural enrichment analysis shows that triacylglycerols were the most significantly enriched chemical class (Fig. 3*C*). Pathway enrichment analysis was unfortunately not possible because exceedingly few (<10%) annotated lipids were associated with KEGG (Kyoto Encyclopedia of Genes and Genomes) identifiers.

We previously identified a positive relationship between the viral load of queens and the presence of supersedure cells within colonies (35), among other variables (see *SI Appendix*, Fig. S2). Given the interrelatedness of queen total viral load, ovary mass, and the presence of supersedure cells, we analyzed the field-sampled queens’ lipidomic data with respect to each variable independently and identified overlapping differential abundance among comparisons. Twenty-three lipids were significantly linked to all three variables (Fig. 3*D*), of which 14 were triacylglycerols (34% of those identified), and two were among the four that were negatively linked to viral load in the cage trial queens (glyceryl trioleate and TG 56:3).

### Ovary Restriction Is Sufficient to Suppress Methyl Oleate Abundance.

**Fig. 4.**
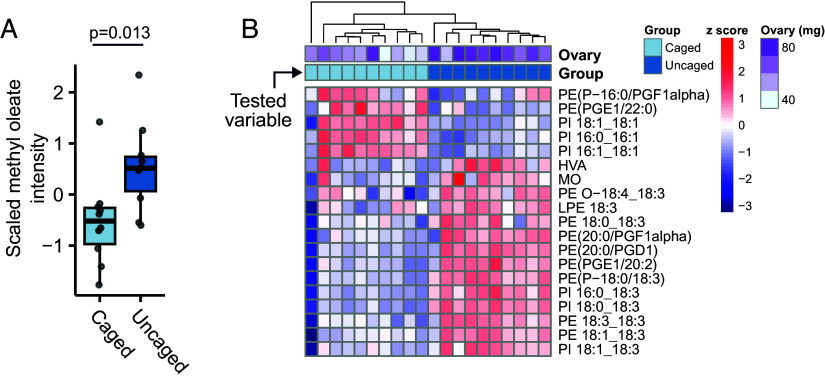
Ovary restriction is sufficient to reduce methyl oleate abundance. We used our previously published lipidomics dataset (55) of in-colony caged (small ovary) and uncaged (large ovary) queens (N = 10 each) to examine how lipids are affected by ovary restriction. (*A*) Scaled methyl oleate abundance in caged and uncaged queens, analyzed using a simple linear model. (*B*) Of the prior 336 lipids annotated in the field samples, 52 were also evaluated in the ovary restriction dataset and 19 of these (shown) were differentially abundant in small- vs. large-ovary queens among a larger pool of 72 differentially abundant annotated lipids (see *SI Appendix*, Fig. S3 for the complete list). Benjamini–Hochberg correction, 5% FDR.

Larger colonies had queens with larger ovaries (*SI Appendix*, Fig. S3), but many confounding factors make the causal direction of this relationship uncertain. One way to manipulate ovary mass independent of other variables is to restrict ovary size via within-colony caging of otherwise equivalent queens (62). We previously used this technique to produce queens with small (caged) and large (uncaged) ovaries (55), and here we capitalized on those samples to determine what lipids, including methyl oleate, are specifically linked to ovary mass. Lipidomics analysis on head extracts from small- and large-ovary queens identified 1,739 molecular features, of which 250 were annotated, including only 13 (5.2%) triacylglycerols (Dataset S1). Seventy-two annotated lipids were differentially abundant in small- versus large-ovary queens (5% FDR, Benjamini–Hochberg method; *SI Appendix*, Fig. S4), including methyl oleate, which was higher in large-ovary queens, as predicted (Fig. 4*A*). Only four triacylglycerols were differentially abundant, representing 31% of those identified, approximately half the fraction significantly linked to virus infection in the virus field trial queens. Two triacylglycerols were upregulated and two were downregulated, in contrast to the concerted downregulation of triacylglycerols with virus infection. Only 52 annotated lipids were detected in both the ovary restriction dataset and the field sample dataset described above, of which 19 (mostly phosphatidylethanolamines and phosphatidylinositols, and no triacylglycerols) were differentially abundant (Fig. 4*B*). Ovary mass restriction via caging is thus sufficient to reduce methyl oleate abundance in addition to changing other lipids (*SI Appendix*, Fig. S4), but poor dataset overlap constrained our ability to compare ovary restriction versus virus effects on the lipidome.

### Ovary Restriction Reduces ApoLP-III But Does Not Affect Immune Proteins.

We previously found that the reduction in ovary mass caused by virus infection is in part a consequence of the cost of immune induction, rather than a direct pathological effect (35). To test whether this trade-off can operate in a bidirectional manner—that is, whether ovary restriction alone is sufficient to conversely elevate immune protein abundance—we assessed immune proteins quantified in the hemolymph of the small- and large-ovary queens (61). We quantified 3,164 unique protein groups in the hemolymph proteome, including all of the immune proteins that we previously found to be associated with virus infection (35), but none significantly varied according to ovary size (5% FDR, Benjamini–Hochberg method; *SI Appendix*, Figs. S5 and S6). A reduction in ovary mass is therefore not sufficient to enable induction of immune protein expression.

Apolipophorin (ApoLP)-III and ApoLP-I/II are major lipid cargo proteins, and ApoLP-III is especially interesting because it functions as a lipid transporter, pathogen recognition protein, and potentiator of immune effectors (see review ref. 28). Given their potential roles as a molecular switch system, we investigated circulating (hemolymph) ApoLP-III (Uniprot accession: B0LUE8) and ApoLP-I/II (A0A7M7SQ18) abundance, as well as the well-known lipoprotein vitellogenin (Q868N5) in small- and large-ovary queens. We found that ApoLP-III abundance was lower in queens with small ovaries (F_1,18_ = 20.9, estimate = −1.43, *P* = 0.00024; *SI Appendix*, Fig. S5*C*), despite immune effectors being unaltered, suggesting that the immune induction activity of ApoLP-III could be attenuated by reducing expression under conditions when resource availability is low. ApoLP-I/II did not differ (F_1,18_ = 0.076, estimate = −0.13, *P* = 0.79), and circulating vitellogenin slightly, but not significantly, declined in queens with larger ovaries (F_1,18_ = 3.6, estimate = −0.80, *P* = 0.074).

To investigate patterns of lipid transporter expression within ovaries, we analyzed three ovary sections approximately corresponding to the germarium (anterior section), early vitellarium (mid-ovary section), and late vitellarium (posterior section) in addition to mature eggs that we sampled from the oviducts. We found that while vitellogenin showed sequentially higher abundance in each successive section (the expected pattern based on progressive accumulation by receptor-mediated endocytosis), ApoLPs did not (*SI Appendix*, Fig. S5*D*). ApoLP-III was significantly less abundant in oocytes compared to late vitellarium sections (linear mixed model, estimate = −1.4, Kenward–Roger df estimate = 27.1, *P* = 0.012), which is consistent with enzyme-mediated delivery of cargo to oocytes [whereby the lipoprotein may still localize to the oocyte surface within ovarioles but is not endocytosed into the egg (19)]. In contrast, ApoLP-I/II abundance increased in mature oocytes (estimate = 1.3, Kenward–Roger df estimate = 26.4, *P* = 0.0018), suggesting that it can become endocytosed and concentrated in eggs. Major lipid trafficking systems in honey bee queens thus appear to be consistent with what is known in other insects.

## Discussion

**Fig. 5.**
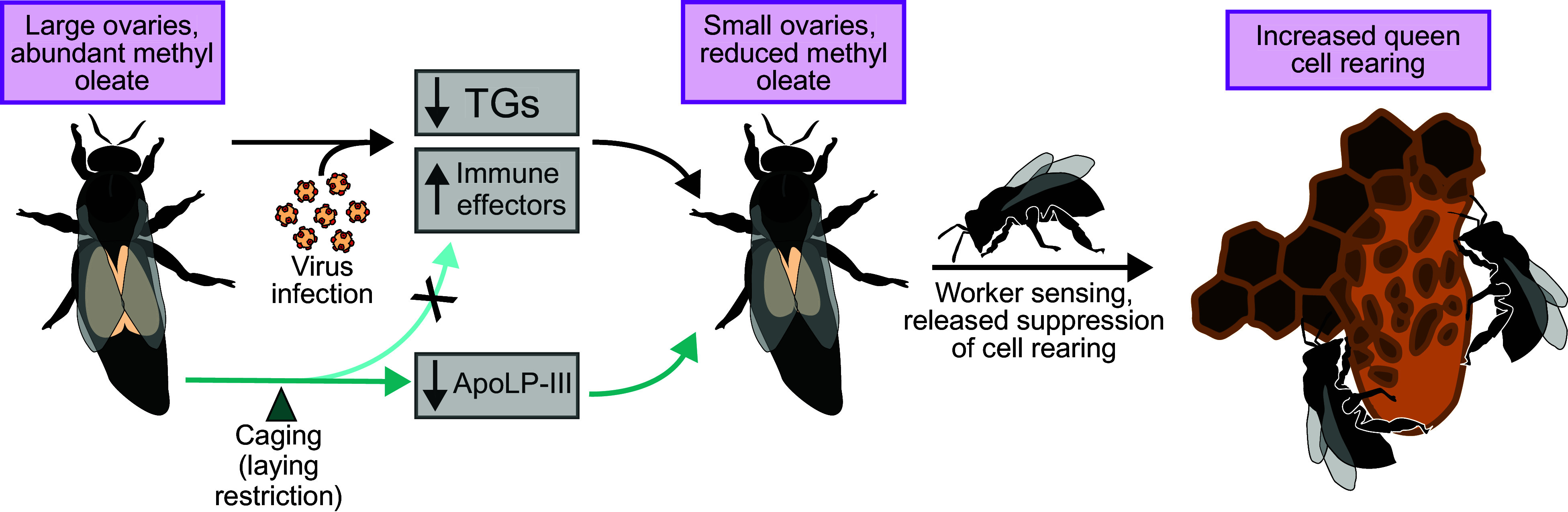
Summary of a candidate supersedure mechanism. Queens with small ovaries produce less methyl oleate, which contributes to worker sensing of compromised queen quality, resulting in increased queen cell rearing. The small-ovary phenotype can arise in multiple ways, such as through virus infection or laying restriction, which coincide with different physiological effects (triacylglyceride, or TG, deficiency and elevated immune effectors in the former, and reduced lipid trafficking in the latter). Dose–response effects of methyl oleate, the relative contributions of other pheromones, and tissue-specific lipidomic shifts deserve further investigation. Image credit: Adapted from ref. 34, which is licensed under CC BY-NC-ND 4.0, and ref. 55, which is licensed under CC BY 4.0.

Here, we show that elevated virus infection perturbs the queen pheromone profile, which is essential for maintaining colony cohesion, by reducing the QRP component methyl oleate in queen heads (Fig. 1). Our field data further demonstrate that methyl oleate contributes to how workers perceive that a queen has become infected and subsequently initiate supersedure (Fig. 2), illustrating a scenario in which virus infection on an individual (the queen) can compromise the well-being of an entire colony. Since ovary restriction alone was sufficient to also reduce methyl oleate abundance (Fig. 4), it may not be the infection, per se, that the workers are sensing; rather, it is more likely the change in ovary investment. These data collectively allow us to draft a working mechanism of supersedure, depicted in Fig. 5. The site of methyl oleate production is not known, but it is present in the hemolymph (12); therefore, an intriguing hypothesis that awaits testing is that the ovaries themselves may release the substance in a manner dependent on their level of egg production.

Other pheromone components are likely also involved in triggering supersedure. For example, the QRP component HVA also declines with ovary mass (55) and tended to decrease with virus infection in this study, but only in the field-sampled queens (and this trend was not significant after multiple hypothesis testing correction), whereas the patterns of methyl oleate abundance were more consistent. Moreover, while the QMP + MO treatment significantly inhibited queen cell rearing and QMP alone (which contains HVA) did not, the latter treatment did tend to suppress cell rearing and is clearly biologically active. These data agree with previous trials where QMP partially, but not entirely, suppressed queen cell rearing (57, 58), and our data suggest that MO is one source of this missing activity. Interestingly, methyl oleate plays diverse roles in reproduction in other insect species (63–65), where its interaction with pathogenic infections has not yet been explored. Additional experiments testing the dose-dependent effects of methyl oleate alone and in combination with additional QRP components, as well as reversibility trials testing methyl oleate’s ability to halt cell rearing that has already commenced, will be necessary to fully understand the relative role of each pheromonal compound in queen rearing inhibition. However, based on the present data, we propose a working mechanism of supersedure stimulation in which elevated virus infection reduces the queen’s ovary mass, which inhibits methyl oleate production, and in turn triggers the workers to initiate queen cell rearing.

High virus infection was linked to broad lipidomic changes in head extracts from field-sampled queens (18% of annotated lipids were differentially abundant), but a comparatively small effect was observed in queens held in the laboratory (only 1% of annotated lipids were differentially abundant). We expected that a stronger effect would be observed in the more controlled laboratory environment; however, since a queen’s lipid intake is influenced by the workers’ royal jelly production, which is itself influenced by the pollen the workers consume (66–68), worker diet differences may have influenced the queen’s overall lipidome. This may also be linked to why such little variation in linolenic acid, which is provided to queens in royal jelly but is originally sourced from pollen (69), was observed in the cage trial queens but not the field samples (Fig. 1). Virus effects in the field may have also been exacerbated by interactions with other stressors or perhaps were only apparent when the queens exceeded a threshold level of ovary activation and metabolic demands (indeed, the ovaries of queens sampled from colonies were 2.3 times larger than those held in the laboratory; *SI Appendix*, Fig. S1). A consistent feature between the two datasets, though, is that methyl oleate and triacylglycerol abundance tended to decline with virus infection (Fig. 3).

Triacylglycerols are one of the densest forms of stored biochemical energy, and this reduction indicates that infections are likely energetically demanding. This is consistent with the presence of our previously observed resource–allocation trade-off with immunity: For a trade-off to occur, a stimulus must first create a resource deficit, and this reduction in triacylglycerols may represent just that (although potential impacts of differential worker feeding behavior to infected queens has not yet been explored). However, extracts from the head are admittedly not the best tissue to analyze whether one were to quantify energy reserves—more relevant would be the fat body—and future work should include comparative lipidomics analysis of multiple tissues to better relate pheromonal and physiological changes. Analysis of additional tissues may also help pinpoint the site of methyl oleate synthesis.

One advantage of conducting lipidomics on head extracts was that the head contains all known components of QRP (12) and analyzing this tissue enabled comparisons with previously published data generated from small- and large-ovary queens (55). We expected queens with small ovaries (laying restricted) to display a reduction in triacylglycerols relative to large-ovary (free laying) queens, mirroring the effect of virus infection (which is itself also an ovary-shrinking stimulus). However, this was not the case; few triacylglycerols (5.2% of all lipids) were identified in the small vs. large ovary dataset compared to the field samples (where triacylglycerols made up 12% of lipids). More striking was the fact that none of those that were differentially abundant were identified in both datasets, and among those in the small- vs. large-ovary dataset, some were upregulated as well as downregulated. Though direct comparisons may not be reliable due to generally poor dataset overlap, it appears that changes in triacylglycerols abundances in small- vs. large-ovary queens does not mirror the effect of virus infection. This could be because laying restriction might not actually cause a resource deficit the way virus infection does. The restricted queens are likely fed less often by the workers, and their ovaries respond by shrinking, but they are also not laying eggs; therefore, resource inputs and outputs may still be balanced in this situation.

Most lipids declined with virus infection, but a select three increased: PGG2, a phospholipid (PEtOH 16:1/16:1), and a sphingolipid (SM 34:0;2O). The significance of the latter two is unclear, but PGG2 is interesting because it is an unstable lipid peroxide that is a precursor to other prostaglandins (70), some of which release oviposition behavior in other insects (71). We did not measure egg laying in the colonies these queens were heading, so we cannot confirm the expected inverse relationship between PGG2 and oviposition (and PGG2 was not identified in the dataset of small- and large-ovary queens), but it is an intriguing possibility. PGG2 was not upregulated in virus-infected queens in our cage trial (Fig. 3*A*), so, if products of its conversion are important for oviposition, they appear only to be relevant in the colony context. PGG2 accumulation also suggests that oxidative stress may be, surprisingly, a collateral cost of laying reduction.

However, this idea is at odds with the fact that the majority of phospholipids downregulated in small-ovary queens contain polyunsaturated fatty acid (PUFA) residues (*SI Appendix*, Fig. S4), which are susceptible to oxidative attack. Therefore, based on PUFA membrane lipid abundances alone, small-ovary, nonlaying queens should be under reduced oxidative stress than large-ovary, laying queens. Laying queens also likely have higher metabolic rates, which would increase reactive oxygen species generation. The overall low frequency of PUFA membrane lipids and low peroxidizability in queens relative to workers has been suggested as one factor enabling queens’ extraordinarily long lives compared to their nonreproductive daughters (72), but the relative importance of PGG2 accumulation vs. reduced PUFA membrane lipid abundance is not yet clear.

Hemolymph proteomics analysis of the small- and large-ovary queens revealed that canonical immune proteins (antimicrobial peptides, lysozyme, and phenoloxidase activating factor) remain unchanged by ovary manipulation (Fig. 5*A*). This indicates that the reproduction–immunity trade-off does not function in reverse (i.e., restricting ovary investment is not sufficient to elevate constitutive immune investment). However, ovary restriction did elevate two unique ferritin proteins (*SI Appendix*, Fig. S5), which are thought to reduce oxidative stress by sequestering iron cations [and thus preventing the formation of hydroxyl radicals (73)] as well as improve immunity by making iron unavailable to pathogens [reviewed in Pham and Winzerling (74)]—roles that are supported by work in bumble bees (75). Whether their main function here is in immunity, mitigating oxidative stress, or both is unknown, but it is noteworthy that these two unique ferritin proteins are upregulated in lockstep in small-ovary queens.

Regardless of the role of these ferritin proteins, ApoLP-III ostensibly does not switch from lipid trafficking to being an immune protein inducer when queens are in an ovary-restricted state (which would enable a bidirectional reproduction–immunity trade-off), as we originally hypothesized. Our previous rationale was that the reduced lipid demands of small ovaries could make more free ApoLP-III available to bind PAMPs and elevate immune effectors downstream of the Toll/Imd signaling pathways, assuming equivalent PAMP abundances. What we observed instead was that circulating immune effector abundance remained the same while ApoLP-III abundance decreased with ovary reduction (Fig. 5). Therefore, it is still possible that our proposed mechanism is correct, but the immune induction capacity of ApoLP-III could be attenuated by downregulating its expression to avoid investing in immune protein expression without an imminent fitness benefit. These lipophorins are clearly tied to queen health and ovary investment but the mechanisms cannot be fully defined without gene knockdown studies or factorially designed experiments with infected and uninfected, caged and uncaged queens. Our ovary section and oocyte protein data do support that ApoLP-III, ApoLP-I/I, and vitellogenin transport lipids in a manner consistent with what is described in other insects (19, 24, 30), though, which forms the foundation for further experimentation.

Importantly, in our previous study on the relationship between queen pheromone profiles and ovary size (55), we reported that the abundance of methyl oleate remained unchanged based on metabolomics-derived peak areas. However, lipidomics data obtained from the same samples through a two-phase extraction demonstrate a clear correlation between methyl oleate abundance and ovary size. This discrepancy likely arises from the presence of coeluting isomers in the metabolomics dataset, which affected the accurate determination of methyl oleate, despite the use of external standards. While the quantification of other compounds in the original metabolomics dataset is not affected, the lipidomics approach used here offers isomeric resolution for analyzing methyl oleate and several hydroxylated fatty acids. This aligns with its established role in queen reproductive signaling and highlights the importance of analytical specificity when analyzing complex lipid mixtures. This serves as a cautionary tale for lipid analysis: Many lipid isomers, particularly those with subtle structural differences (e.g., double-bond positioning or branching), are not routinely resolved by shotgun lipidomics or traditional methods (76). These isomers are often absent from literature databases and may coelute in standard workflows, leading to misinterpretations of lipid abundance and function. Our outcomes highlight the necessity of isomer-sensitive techniques when studying lipids in complex biological systems. Overlooking these nuances risks missing critical biological insights, especially in contexts where lipid structural diversity drives physiological outcomes.

## Conclusion

These data highlight a process by which high levels of virus infection can cause physiological changes in an individual that profoundly affect the trajectory of a colony. Our goal here was to answer five core questions pertaining to queen–virus interactions: 1) does elevated virus infection perturb QRP profiles and does this play a role in stimulating supersedure cell rearing, 2) what other lipids are affected by virus infection, 3) is ovary restriction alone sufficient to alter QRP and lipids, 4) does ovary restriction cause a reverse reproduction–immunity trade-off (i.e., are immune effectors elevated under ovary restriction), and 5) could ApoLP-III be mediating the reproduction–immunity trade-off induced by virus infection? We have answered all these questions with varying degrees of certainty. Elevated virus infection reduces methyl oleate abundance as well as many triacylglycerols, indicating a change in pheromone signaling in concert with a depletion of stored energy. Since methyl oleate is necessary to suppress queen cell rearing, our data strongly suggest that this pheromonal perturbation triggers workers to initiate supersedure. Ovary restriction also reduces methyl oleate abundance (but not triacylglycerols); therefore, the impact of virus infection on methyl oleate is an indirect effect of diminished ovaries. Since canonical immune proteins do not increase under ovary restriction, the reproduction–immunity trade-off only operates under immune stimulation and not restricted laying. Our data are consistent with positioning ApoLP-III as a candidate molecular switch that could govern reproduction vs. immunity investment, but more detailed experimentation is necessary to assert this claim. We present a plausible mechanistic pathway by which high virus infections reduce queen ovary function and disrupt pheromone signaling, thereby triggering worker-driven queen replacement—a process with broad implications for the social regulation of reproduction across eusocial insects.

## Methods

### Cage Trial and Field Sample Queens.

The samples described here were derived entirely from cage trials and field samples that have been previously described (35), but for which pheromone and lipidomic data had not been acquired. Briefly, in the cage trial, young (< 1 mo old) queens were microinjected with either saline, a live virus inoculum (a mix of 100 million BQCV copies and 2 million DWV-B copies, to approximate common co-occurrence levels of these viruses in queens), or a UV-inactivated virus inoculum (the same concentration of virus particles as the live inoculum, but that were inactivated by UV-crosslinking), with n = 9 queens in each group. The queens were housed in queen monitoring cages (77) with ~100 young workers as well as ad libitum fondant, pollen substitute, and water. The cage walls were made of replaceable egg-laying plates to facilitate laying. The queens were observed for 7 d after injection to monitor egg laying, then were killed, dissected, and samples were stored at −70 °C. Infected queens were confirmed by RT-qPCR to have significantly higher BQCV and DWV-B loads compared to controls. Here and elsewhere, additional nonviral pathogens (e.g., *Nosema*) were not tested, but are expected to be randomly distributed among groups since in all experiments queens were age- and batch-matched. See Chapman et al. (35) for complete experimental details, including RT-qPCR viral analyses.

The field samples are derived from age-matched queens (from the same source as the cage trial) heading 32 five-frame nucleus colonies, as previously described (35). Colonies were located in two different apiaries (sites) in a balanced design, and all colonies received ad libitum pollen substitute (15% pollen patties, Global) and Hive Alive fondant for the duration of the experiment. The queens were blindly divided into two groups (n = 16 each), with one receiving a saline injection and the other receiving the same live virus inoculum described above. However, upon completion of the trial (7 wk after injection) RT–qPCR analysis showed that the queens receiving the live virus inoculum did not have higher viral loads than the saline controls; rather, the queens’ viral abundances correlated significantly with worker viral abundance (*SI Appendix*, Fig. S2). We therefore treated the experiment as an observational dataset, correlating queen virus abundance to traits of interest irrespective of the experimental group. Queens were euthanized and stored as above. See Chapman et al. (35) for complete experimental details, including viral analyses.

### Pheromone Field Trial.

To determine the effect of methyl oleate on queen cell rearing suppression, we produced n = 30 queenless 4-frame nucleus colonies and manipulated the pheromones we added back. The colonies were produced from the same stock of bees (originating from Quebec, Canada) at the same apiary by dequeening 15 8-frame colonies (none of which showed signs of supersedure cell rearing) and splitting them in half in situ, ensuring that each resulting 4-frame unit contained all the resources necessary to rear a new queen (abundant honey, available pollen, eggs and newly hatched larvae, and an abundant worker population). Then, we randomly assigned the colonies to belong to one of three groups with n = 10 colonies each: control (no pheromone added), QMP + solvent, or QMP + methyl oleate. The QMP + solvent exposures were achieved by placing one TempQueen (Intko Supply Ltd., Chilliwack, BC) pheromone strip, to which 8 µL of 80% ethanol solvent was added and allowed to dry, in the center of each colony. These plastic strips are impregnated with the five QMP components 9-ODA, 9(R)-HDA, 9(S)-HDA, HVA, and HOB, but not methyl oleate. The QMP + methyl oleate exposures were achieved by pipetting 80 µg of methyl oleate dissolved in 8 µL of 80% ethanol onto each pheromone strip and allowing the solvent to evaporate, then placing each strip in the center of each colony. This dose corresponds to approximately 10 times one queen equivalent per day of the trial, and was chosen based on the observation that 3 to 4 µg of methyl oleate is extractable from a queen (12), but the substance is presumably under constant production and removal by worker contacts. Given the unknown rate of production and transfer to workers combined the variable abundance within queens, we selected this higher dose for our single-application, multiday trial. To maintain blindness, an operator not involved in further colony assessments added the pheromone strips and labeled the colonies under a pseudonym such that the identity of QMP + solvent and QMP + methyl oleate groups were concealed. Colonies were left undisturbed for 52 h, then each frame was inspected for the presence of active queen cells, i.e. queen cells that were either a) charged with royal jelly and a larva, b) containing an egg, or c) primed with royal jelly but no visible egg or larva. The level of commitment to cell rearing is different in these three situations, with the former being the highest and the latter two less so; therefore, we analyzed the data considering four colony-level dependent variables: 1) queen cell score, 2) number of charged queen cells, 3) number of active queen cells, and 4) presence or absence of active queen cells. The queen cell score was calculated by weighting charged queen cells more heavily than other cell types, such that cells charged with royal jelly and a larva were assigned a score of 2, cells containing an egg or royal jelly priming were assigned a score of 1, and all scores were added together for each colony (e.g., a colony with three charged queen cells and one cell with an egg would have a score of 7). The number of charged queen cells included only cells charged with royal jelly and a larva, ignoring cells with an egg or primed with royal jelly, while the number of active queen cells included all three types of cells, and the presence or absence of active cells was represented by a binomial variable (1 = at least one active cell present in the colony, 0 = no active cells).

### Statistical Analysis of Pheromone Exposure Data.

We used R (version 4.3.0) via R Studio (version 2023.09.1 + 494) (78) to assess the effect of experimental group (control, QMP + solvent, or QMP + methyl oleate) on each response variable separately. We used generalized linear mixed models to assess the impact of group on queen cell score, number of charged queen cells, and number of active cells, including parent colony origin as a random intercept and specifying a Poisson distribution. To model the presence or absence of active cells, we used the same model structure, specifying a binomial distribution. In all cases, we used tools within the DHARMa (79) package to confirm appropriateness of residual distributions, and post hoc testing was conducted using emmeans (80) with the Tukey *P* value adjustment method applied.

### Queens Sampled for Ovary Restriction Analysis.

The queens sampled and analyzed for the ovary restriction experiment have been previously described (55). Briefly, queens were reared simultaneously at a single queen production operation from a single mother colony. Queen cells were placed in mating nucleus colonies and once emerged, the queens were allowed to freely mate. 2 wk after emergence, a cohort of mated queens were placed in JZBZ queen cages, which are large enough to house the queen but do not support egg laying, and placed in a “queen bank” (a large colony with sufficient young bees to feed the queens through the cage screen). 1 mo after emergence, n = 10 of these queens were sampled, making up the “small ovary” (caged) group. Another n = 10 queens that had remained laying in their nucleus colonies were sampled on the same date, making up the “large ovary” (uncaged) group.

### Queens Sampled for Ovary Section Analysis.

To compare lipid transporter abundance in ovary sections, we dissected queens acquired from a routine apiary requeening sweep and divided ovaries into three parts with a scalpel as illustrated in *SI Appendix*, Fig. S6. Queens originated from a single batch of Northern Californian imports that headed colonies and overwintered in a single apiary, then were allowed to grow in size naturally through the late winter and early spring. Queens were then sampled in April, transported to the laboratory in queen cages with 5 attendant workers and queen candy, then anesthetized (5 min carbon dioxide exposure), decapitated, and dissected within 2 h of being removed from colonies. Ovaries were divided into sections under a dissection microscope and mature oocytes were delicately removed from the lateral oviducts, then proteins were extracted from tissues exactly as previously described (81).

### Lipid Extraction and LC–MS/MS Analysis.

Lipids (including QRP components) were extracted from cage trial and field sample queen heads using a two-phase extraction procedure (82) and analyzed by LC–MS/MS exactly as previously described (55). Heads were chosen because this is the only body segment known to contain all QRP compounds (12), and this tissue enables comparisons to previously published data (55). See supplementary methods within *SI Appendix* *f*or detailed information, including sample processing, LC–MS/MS parameters, and data processing steps.

All mass spectrometry data are housed at Metabolomics Workbench (www.metabolomicsworkbench.org). Data derived from cage trial and field sample queens were newly acquired (doi: 10.21228/M88838; project PR002316) (83), whereas data from ovary-restricted queens were previously published (doi: 10.21228/M81B11; project PR001924) (55, 84). Annotated lipid abundance data are also available in Dataset S1.

### Statistical Analysis of Lipidomics Data.

Peak areas corresponding to the quantifiable components of the queen retinue pheromone (9-ODA, 9(R)-HDA, HVA, HOB, LEA, MO, and CA) were identified with high confidence by comparing spectra and retention times to external standards. These a priori compounds of interest were analyzed separately from the complete lipidomics dataset. We lacked an external standard for 9(S)-HDA; therefore, only the R enantiomer was evaluated. PA (palmityl alcohol), which is also a QRP component (12), occurred below our limit of detection; therefore, seven of the nine QRP components were assessed here. See McAfee et al. (55) for vendor sources of external standards.

We used R (version 4.3.0) via R Studio (version 2023.09.1 + 494) for all subsequent statistical analyses (78). Queen and colony metadata (virus abundances, ovary masses, presence of supersedure cells, etc.) are publicly available from our previous publication (35). We modeled each of the seven QRP components individually using either additive queen virus load or ovary mass as a predictor in a simple linear model. Appropriateness of model fit was confirmed by inspecting residual distributions. A Bonferroni correction (α = 0.05/7 = 0.0071) was applied.

In our previously published results (35), we reported that queen total viral load was a negative but not significant predictor of ovary mass, and that total viral load was a marginally nonsignificant predictor of supersedure cells; however, there were inconsistencies in the way that total viral load was calculated, and in both cases the relationships are actually significant. In the prior study, copy numbers (per ng of RNA) were first transformed (natural logarithm) then summed, but this approach biases the total viral load metric by weighting queens infected with multiple viruses more heavily, instead of reflecting the simply summed copy numbers, as we have previously done (33, 85). To correct this error and regain consistency with our previous studies, we recalculated total viral load by first summing copy numbers per ng RNA, then log10 transforming, and reanalyzed worker viral load as a predictor of queen viral load (linear model), queen viral load as a predictor of ovary mass (linear model), and queen viral load as a predictor of supersedure cell presence (generalized linear model). The results of these reanalyses are shown in *SI Appendix*, Fig. S2. In the present study, we use the corrected method of calculating total viral load in all instances.

To assess lipidomic changes, we scaled and combined all annotated lipids identified in positive and negative ion mode into a single dataset of 336 compounds. We log10 transformed the data and visually confirmed normality of the overall data distribution. We used tools within the limma package (86), which enables empirical Bayes variance estimation, to identify significant relationships between lipid compound abundances and total virus load (continuous predictor) for both the cage trial (final n = 27) and field sample data (n = 29, down from 32 due to three instances of queen rejection, supersedure, or mishandling). The field sample lipidomics data were additionally analyzed with respect to the presence or absence of supersedure cells in the colony (categorical predictor, two levels), and, separately, ovary mass (continuous predictor). In all cases, false discovery rates were controlled to 5% using the Benjamini–Hochberg method. Because total virus load predicts both ovary mass and the presence/absence of supersedure cells, including both variables in the same model would obscure our ability to detect potentially meaningful relationships. Therefore, we instead analyzed the data with respect to each variable separately and compared the overlap among significantly different lipids. To compare abundance of specific lipids between two groups, we used simple linear models and inspected residual distributions to confirm appropriateness of fit.

### Structural Enrichment of Lipid Classes.

We used ChemRich (87) to detect statistical enrichment of lipid structural classes with respect to *P* values associated with total viral load. We obtained CIDs, SMILES, and InChiKeys for each lipid compound from the PubChem database (https://pubchem.ncbi.nlm.nih.gov/). For instances where the positions of double bonds or functional groups were unknown, we assumed the compound was one of the available isomers not already represented in the dataset. This approach was justified because enrichment analysis of structural classes depends on the presence, rather than the specific positions, of functional groups or unsaturation; therefore, these ambiguous isomers are best handled by still representing them in the data. Significance of enrichment was controlled to 5% FDR using the Benjamini–Hochberg method.

### Proteomics Data Acquisition and Data Processing.

To assess the impact of ovary restriction on immune effector and lipid transporter expression, we analyzed publicly available hemolymph proteomics data from ovary restricted and unrestricted queens (61) housed on the MassIVE proteomics data archive (https://massive.ucsd.edu/ProteoSAFe/static/massive.jsp) under accession MSV000094277. These data were partially analyzed in the associated publication, but data from the caged queen cohort were not presented as we were previously focused on assessing queen aging in their natural (unrestricted) environment. All samples were processed in parallel with those previously described and the data acquisition and processing methods are identical (61). These are the same ovary restricted queens as described in the lipid analysis section (lipids were extracted from their heads and proteins were extracted from their hemolymph). Proteomics methods for the ovary section samples can be found in the *SI Appendix*, *Supplementary methods*. The protein quantification data are supplied in Dataset S1.

### Statistical Analysis of Proteomic Data.

Proteome-wide differential expression analysis of the small- and large-ovary queen hemolymph was conducted using the limma package (86) essentially as described for the lipidomics data analysis. The exceptions are that the data were first filtered to remove contaminant protein sequences and proteins identified in fewer than 50% of the samples. FDR was controlled to 5% using the Benjamini–Hochberg correction. Specific proteins of interest (vitellogenin, ApoLP-III, and ApoLP-I/II) were further analyzed using a simple linear model to compare abundance between two groups (small-ovary vs. large-ovary queens). The abundance of these proteins in the independent ovary section dataset were assessed using linear mixed models to compare abundance in ovary sections (four levels: germarium, early vitellariums, late vitellarium, and germarium). The mixed model also included queen as a random effect (10 levels) to account for nonindependence of samples.

## Supplementary Material

Appendix 01 (PDF)

Dataset S01 (XLSX)

## Data Availability

Lipidomics data have been deposited in Metabolomics Workbench (10.21228/M88838 and 10.21228/M81B11) (83, 84).
