# Supplementary material for: Elevated virus infection of honey bee queens reduces methyl oleate production and destabilizes colony-level social structure
Source: Proc Natl Acad Sci U S A. 2025 Oct 14;122(42):e2518975122. doi: 10.1073/pnas.2518975122 (PMC12557728; doi:10.1073/pnas.2518975122)
Supplement: Supplementary file 1 — Appendix 01 (PDF) [file pnas.2518975122.sapp.pdf]

Elevated virus infection of honey bee queens reduces methyl oleate production and destabilizes colony-level social structure

Alison McAfee[1,2]*, Abigail Chapman[1], Armando Alcazar Magaña[1,3], Katie E. Marshall[4], Shelley E. Hoover[5], David R. Tarpy,[2] Leonard J. Foster[1,3]**

1. Michael Smith Laboratories, Department of Biochemistry and Molecular Biology, University of British Columbia, Vancouver, BC, Canada
2. Department of Applied Ecology, North Carolina State University, Raleigh, NC, USA
3. Life Sciences Institute, Department of Biochemistry and Molecular Biology, University of British Columbia, Vancouver, BC, Canada
4. Department of Zoology, University of British Columbia, Vancouver, BC, Canada

5. Department of Biological Sciences, University of Lethbridge, Lethbridge, AB, Canada

*Correspondence: alison.n.mcafee@gmail.com

**Correspondence: foster@msl.ubc.ca

Supplementary figures

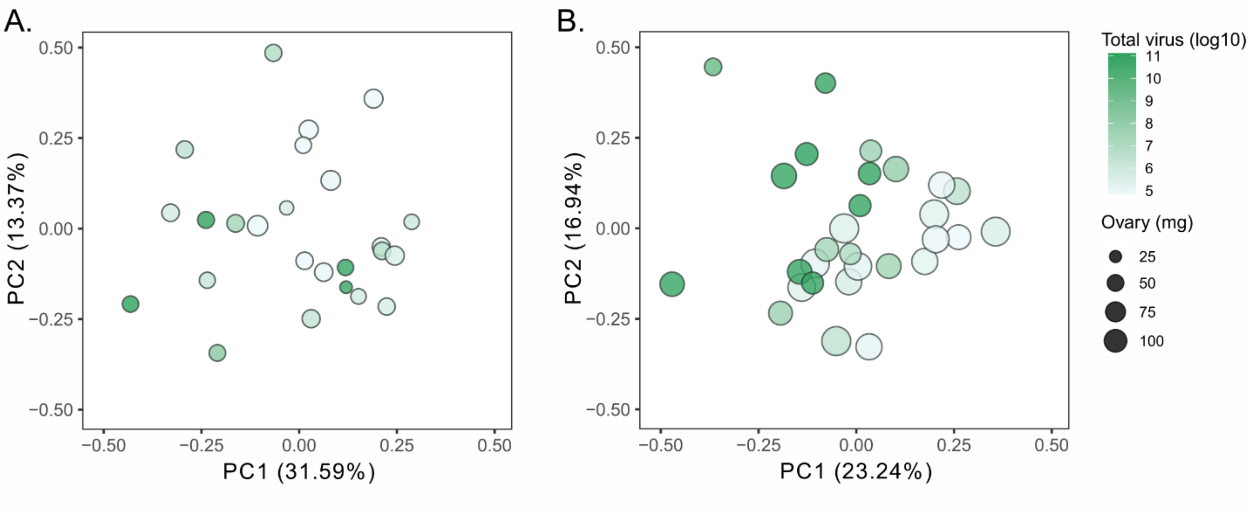

Figure S1. Principle component analysis suggests that lipid profiles are linked to viral loads in the field. Lipidomics analysis of queen head extracts identified 337 annotated compounds overall. A) Cage trial data (N = 27 queens). N = B) Field sample data (N = 29 queens). As previously reported (1), ovary masses among cage trial queens were significantly smaller than field trial queens due to reduced egg laying ability in the miniature laboratory laying cages.

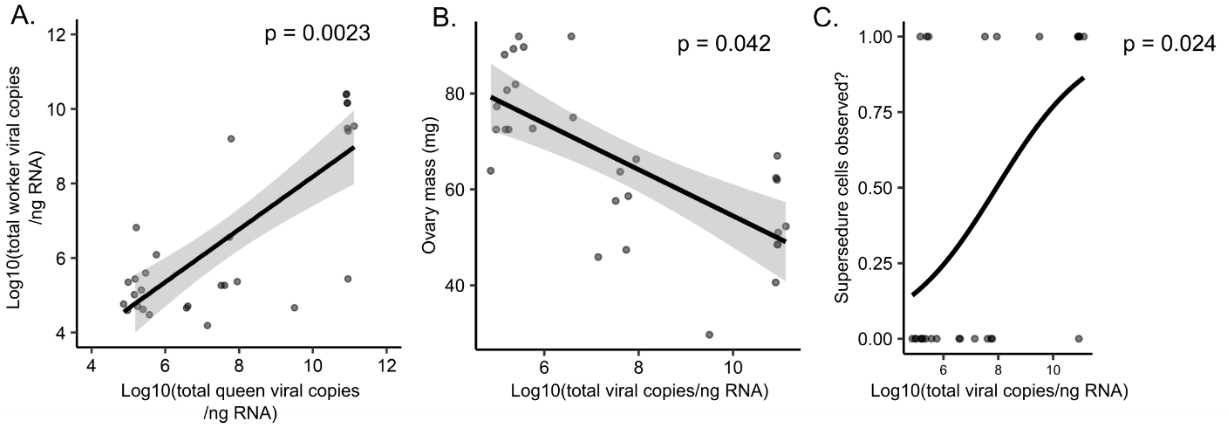

Figure S2. Corrections to prior analyses of queen virus load. We reanalyzed previously published data, correcting the manner by which total viral load metrics were calculated. Previously, raw virus copy numbers were log transformed, then summed to produce the "viral load" variable; however, this leads to unjustified weighting of multiplexed infections. "Viral load" is likely better represented by summing virus copy numbers prior to transforming. When analyzed in this way, our previous conclusions (1) are strengthened. A) Worker viral load positively predicted queen virus load (linear model; $F_{2,26} = 39.9$, estimate = 0.47, p = 0.0023). B) Queen virus load negatively predicted queen ovary mass (linear model; $F_{2,26} = 14.0$, estimate = -3.3, p = 0.044). C) Queen virus load positively predicted the presence of supersedure cells in colonies (1 = present, 0 = absent; generalized linear model; estimate = 0.77, df = 26, p = 0.025). In all models, apiary site (categorical predictor, two levels) was also included as a predictor.

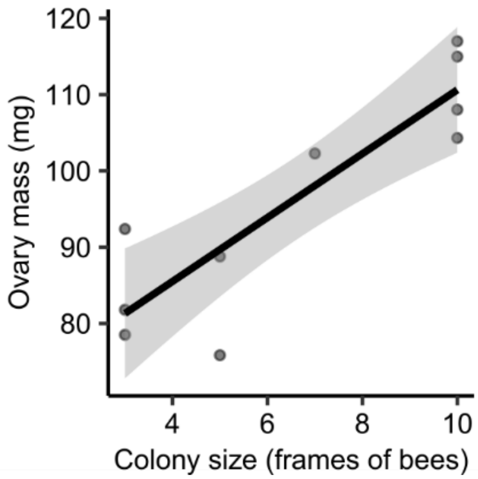

Figure S3. Queen ovary mass is positively linked to colony size. Honey bee queens (n = 10, Northern Californian origin, 1 y old, overwintered) were sampled during routine apiary requeening in the spring (April) at the University of British Columbia. At the time of sampling, colonies were scored for their size, where 1 frame of bees equals a visual estimation of ≥75% adult bee area coverage of the frame. Wet ovary mass was measured using an analytical balance. The relationship was significant and positive (linear model, $F_{1,8} = 28.4$, estimate = 4.2, p = 0.00071).

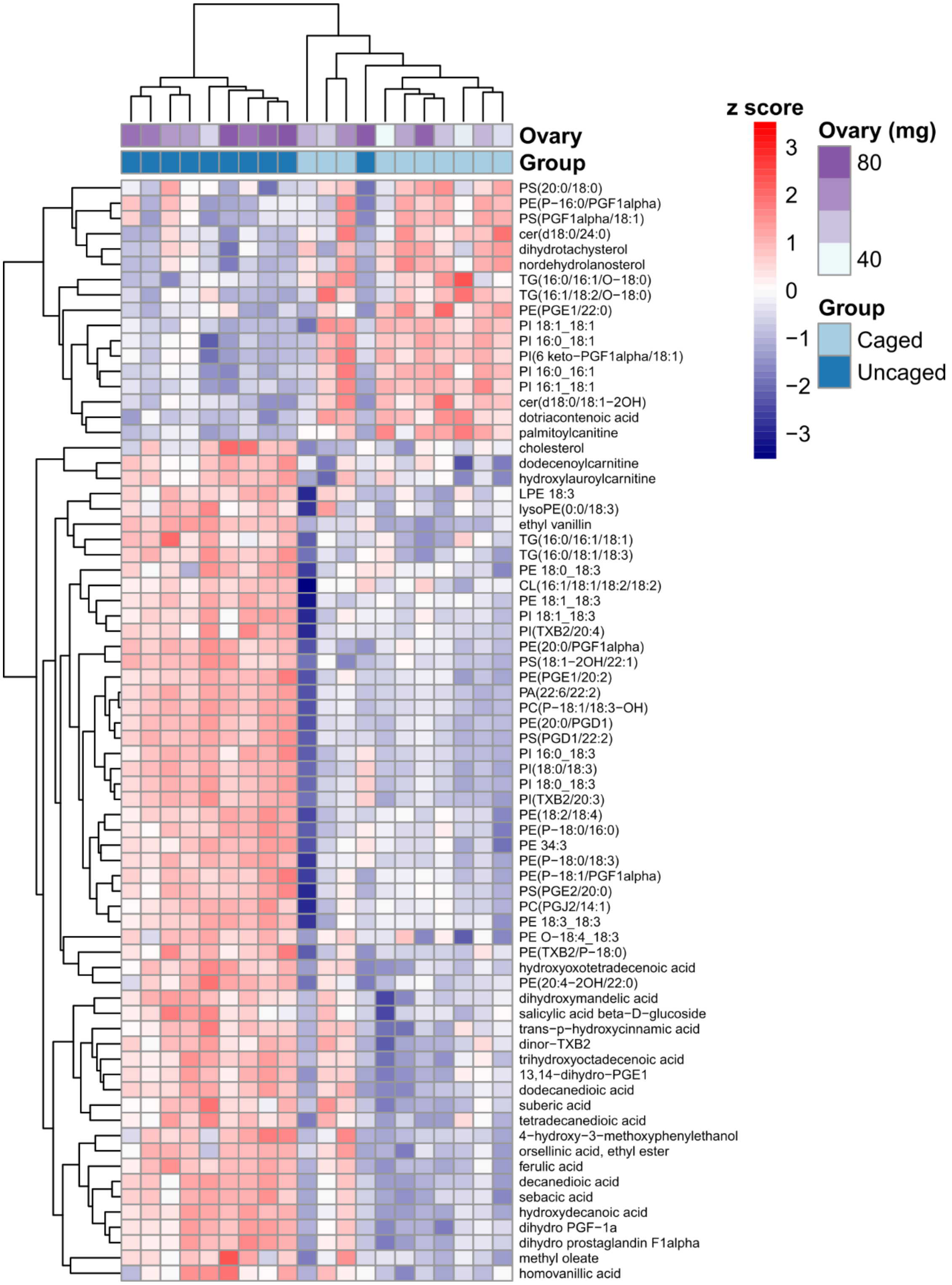

Figure S4. **Laying restriction alters abundance of annotated lipids from queen head extracts.** Lipids (72) significantly different in small-ovary (caged; n = 10) versus large-ovary (uncaged; n = 10) queens. FDR = 5% (Benjamini-Hochberg method).

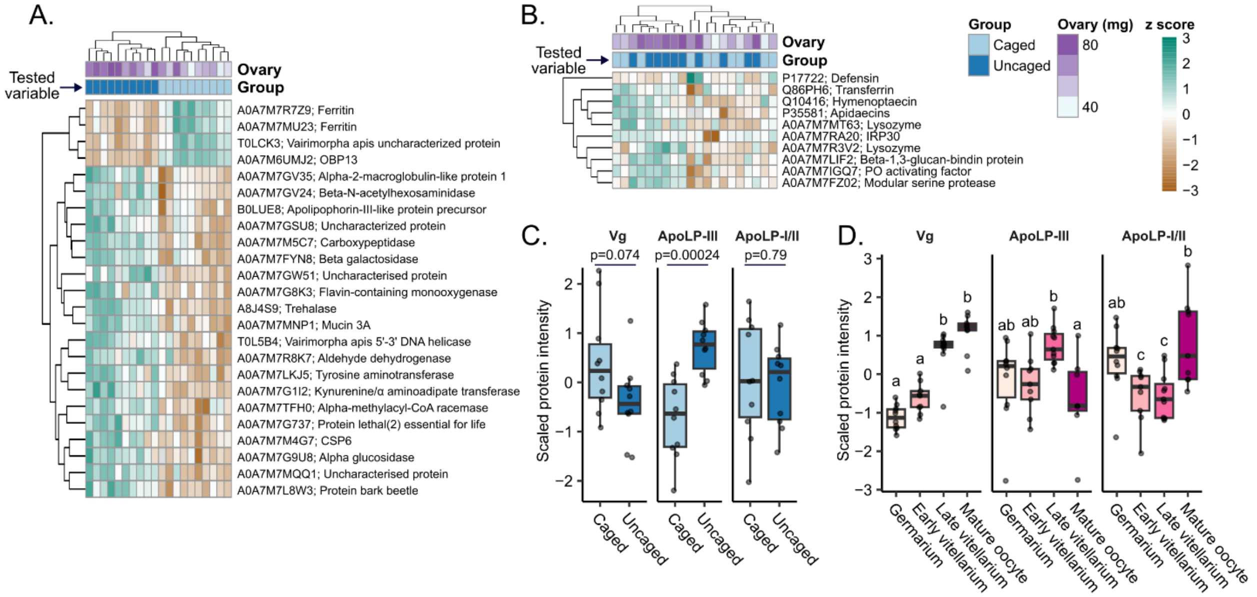

Figure S5. Hemolymph protein analysis in caged versus uncaged queens. A) All differentially expressed proteins in small- (caged; N = 10) vs. large-ovary (uncaged; N = 10) queens (5% FDR, Benjamini-Hochberg correction). Ovary restriction affects ApoLP-III but is not sufficient to alter expression of circulating immune effectors. B) Immune effectors that were previously found to be stimulated by virus infection (1) are not significantly linked to ovary size (5% FDR, Benjamini-Hochberg correction). C) Analysis of specific circulating lipid transporters quantified in hemolymph of small- vs. large-ovary queens. P values indicate results of t-tests. Vg = vitellogenin; ApoLP-III = apolipophorin-III-like protein; ApoLP-I/II = apolipophorins-I/II. D) Lipid transporters quantified in progressive ovary sections (approximating the germarium, early vitellarium, and late vitellarium) and mature oocytes collected from the lateral oviduct. Different letters indicate statistical significance between groups, as determined by linear mixed modelling with section as a fixed effect (4 levels) and queen as a random effect (10 levels). Pairwise comparison p values were corrected by the Tukey method.

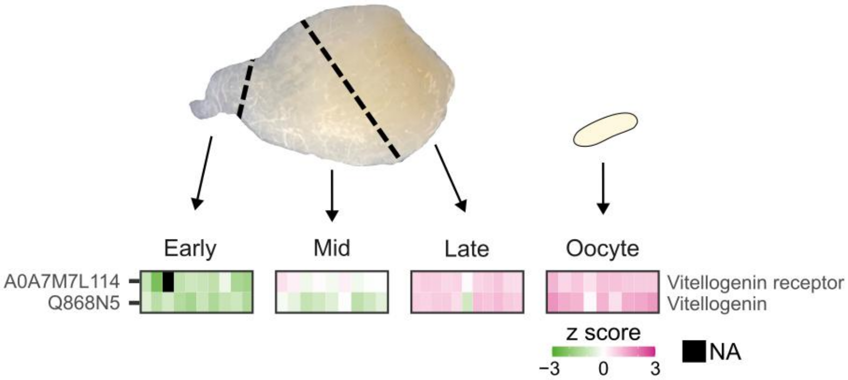

Figure S6. **Example ovary dissection diagram for assessing expression of proteins linked to lipid transport.** Approximate sections: Early = germarium, Mid = early vitellarium, Late = late vitellarium; Oocyte = mature oocyte retrieved from lateral oviducts. Vitellogenin and vitellogenin receptor shown as example expression patterns.

Supplementary methods

Lipid extraction

The queen heads were homogenized in methanol extraction solvent (400 µl 75% methanol, 0.1% butylated hydroxytoluene, in ddH_2O), then methylated tert-butyl ether (1 mL) was added and the samples were incubated for 1 h (1000 rpm shaking, room temperature). Next, clarified extract was mixed with water to induce phase separation. After a brief incubation at room temperature (10 min), samples were centrifuged (14,000 g, 15 min, 4 °C) and 250 µl of the upper phase (lipids) was retained for lipidomics. We used a combination of internal and external standards to facilitate peak identification (see McAfee *et al*. (2) for complete details). The samples were stored at -70 °C in solution until LC-MSMS analysis.

Lipidomics LC-MS/MS analysis

Lipidomics analysis was conducted as previously described (2). Lipid extracts were resuspended in a solution of 70% acetonitrile and 30% isopropanol, and separated using a Vanquish UHPLC system (Thermo) equipped with an ACQUITY UPLC CSH C18 analytical column (130 Å, 1.7 µm, 2.1 mm × 100 mm, Waters). A multi-step gradient was employed, increasing from 20% to 99% mobile phase B over 18 minutes. Mobile phase A consisted of water, while mobile phase B was a mixture of acetonitrile and isopropanol, both containing 10 mM ammonium formate and 0.1% formic acid. The column was maintained at a temperature of 65°C with a flow rate of 0.4 mL/min, and the samples were kept at 4°C. Mass spectrometry (MS) analysis was performed on a Bruker Impact II QTOF in both positive (ESI+) and negative (ESI−) electrospray ionization modes. The capillary voltages were set to +4,500 V for ESI+ and -3,800 V for ESI−, with a dry gas temperature of 220°C, and a scan range of 100–1,700 m/z. To enhance sensitivity for hydroxy fatty acids and improve structural coverage, additional improvements were

implemented for the ESI+ dataset: the spectral acquisition rate was set to 2 Hz, and a stepped MS/MS collision energy varying from 100% to 250% was applied, with 50% timing at each energy level. Dynamic MS/MS acquisition was utilized, targeting an intensity of 1125 counts, with an acquisition frequency ranging from a minimum of 20 Hz to a maximum of 40 Hz. Finally, structural information was improved by implementing a 15-second active exclusion window, minimizing redundant fragmentation and improving isomer coverage.

Lipidomics data processing

Annotations were conducted following the Metabolomics Standards Initiative (MSI) reporting criteria, as previously detailed (3). The raw data were processed using Progenesis QI software (version 3.0.7600.27622) with the METLIN plugin (version 1.0.7642.33805, NonLinear Dynamics). This process included peak picking, alignment, deconvolution, normalization, and database querying. Level 2 annotations were assigned by screening features against databases such as METLIN (4), GNPS (5), HMDB (6), MassBank of North America (7), and LipidBlast (8). For compounds detected in both ion modes, the compound with lower variability in quality controls (QCs) was retained.

Proteomics analysis of ovary section samples

Proteomics data for the ovary section samples were acquired by injecting 250 ng on an Easy nLC-1000 system (Thermo) connected in-line to an Impact II QTOF mass spectrometer (Bruker). Liquid chromatography and data acquisition methods were exactly as previously described (9). Raw data were searched using MaxQuant (10) version 1.6.1.0 with default search options except that match between runs was enabled. The data were searched against the honey bee reference proteome available on NCBI (based on the build HAv3.1, downloaded Nov 18th, 2019) with all honey bee virus and Nosema (*Vairimorpha*) proteins included in the FASTA file.

References:

1. A. Chapman *et al.*, Common viral infections inhibit egg laying in honey bee queens and are linked to premature supersedure. *Scientific Reports* **14**, 17285 (2024).
2. A. McAfee, A. A. Magaña, L. J. Foster, S. E. Hoover, Differences in Honey Bee Queen Pheromones Revealed by LC-MS/MS: Reassessing the Honest Signal Hypothesis. *iScience* (2024).
3. L. W. Sumner *et al.*, Proposed minimum reporting standards for chemical analysis: Chemical analysis working group (CAWG) metabolomics standards initiative (MSI). *Metabolomics* **3**, 211-221 (2007).
4. C. Guijas *et al.*, METLIN: A technology platform for identifying knowns and unknowns. *Analytical Chemistry* **90**, 3156-3164 (2018).
5. M. Wang *et al.*, Sharing and community curation of mass spectrometry data with Global Natural Products Social Molecular Networking. *Nature Biotechnology* **34**, 828-837 (2016).
6. D. S. Wishart *et al.*, HMDB 5.0: The human metabolome database for 2022. *Nucleic Acids Research* **50**, D622-D631 (2022).
7. H. Horai *et al.*, MassBank: a public repository for sharing mass spectral data for life sciences. *Journal of Mass Spectrometry* **45**, 703-714 (2010).

8. T. Kind *et al.*, LipidBlast in silico tandem mass spectrometry database for lipid identification. *Nature Methods* **10**, 755-758 (2013).
9. A. McAfee, B. N. Metz, J. P. Milone, L. J. Foster, D. R. Tarpy, Drone honey bees are disproportionately sensitive to abiotic stressors despite expressing high levels of stress response proteins. *Communications Biology* **5**, 141 (2022).
10. J. Cox *et al.*, Accurate proteome-wide label-free quantification by delayed normalization and maximal peptide ratio extraction, termed MaxLFQ. *Molecular and Cellular Proteomics* **13**, 2513-2526 (2014).
